# Electrophysiological characterization of schizophrenia-associated variants in NaV1.2 sodium channel

**DOI:** 10.1192/j.eurpsy.2022.1967

**Published:** 2022-09-01

**Authors:** M. Suslova, P. Hautvast, A. Gaebler, A. Lampert

**Affiliations:** 1 Uniklinik RWTH Aachen, Institute Of Physiology, Aachen, Germany; 2 Faculty of Medicine, RWTH Aachen, Department Of Psychiatry, Psychotherapy And Psychosomatics, Aachen, Germany

**Keywords:** schizophrénia, Electrophysiology, site-directed mutagenesis, Voltage-gated sodium channels

## Abstract

**Introduction:**

A major pathophysiological hypothesis of schizophrenia states an increased activity of glutamatergic neurons leading to an imbalance of neural excitation and inhibition (E/I-imbalance). One potential molecular mechanism of E/I-imbalance is a dysfunction of voltage-gated sodium channels, which are crucial for the generation of action potentials, the fundamental event of neuronal excitation. Indeed, patients with schizophrenia exhibit an increased burden of rare exonic variants of sodium channel genes, but the literature describing their electrophysiological effect is scarce.

**Objectives:**

The aim of this project is to assess the functional impact of three mutations of the Sodium Voltage-Gated Channel Alpha Subunit 2 (SCN2A) gene / Na_V_1.2 channel which were identified in four patients with schizophrenia, using a heterologous expression system.

**Methods:**

Three variants of the human SCN2A gene (R850P, V1282F and S1656P) were created using site-directed mutagenesis. HEK293T cells transfected with either the mutant or wild type constructs are being investigated by voltage-clamp technique, applying activation, steady-state fast inactivation, use dependency and ramp protocols.

**Results:**

All three mutated constructs were successfully created. Preliminary recordings from the V1282F mutant indicate a shift of both the activation and steady-state fast inactivation to the hyperpolarized direction.

**Conclusions:**

In a subgroup of patients, E/I imbalance may be a consequence of Nav1.2 mutations leading to increased excitability of glutamatergic neurons. By integrating insights from different mutations we aim to identify traits of a potentially shared disease pathway which may provide a basis for the development of novel therapeutics.

**Disclosure:**

No significant relationships.

